# Impact of psychosocial status on sleep quality in adolescents with childhood-onset systemic lupus erythematosus

**DOI:** 10.1186/s12969-026-01216-5

**Published:** 2026-05-13

**Authors:** Sonya Mar‘atusalihat, Reni Ghrahani, Rodman Tarigan

**Affiliations:** Department of Child Health, Faculty of Medicine, Universitas Padjadjaran, Hasan Sadikin General Hospital, Pasteur 38, Bandung, West Java 40161 Indonesia

**Keywords:** Childhood-onset systemic lupus erythematosus, Adolescents, Psychosocial status, Depression, Sleep quality

## Abstract

**Background:**

Adequate sleep is essential for neurodevelopment and immune regulation, yet adolescents with childhood-onset systemic lupus erythematosus (cSLE) are at high risk for sleep disturbances. While these associations are well-documented in adults, data on the pediatric population remains limited. This study evaluated sleep quality in Indonesian adolescents with cSLE and examined its association with psychosocial distress.

**Methods:**

This cross-sectional study enrolled 60 adolescents (aged 10–18 years) diagnosed with cSLE at a tertiary hospital in Bandung, Indonesia. Psychosocial status was screened using the Pediatric Symptom Checklist-17 (PSC-17), and sleep quality was assessed via the Pittsburgh Sleep Quality Index (PSQI). Disease activity was measured using the MEX-SLEDAI. The data were analyzed using Chi-squared or Fisher’s exact tests as appropriate and Spearman correlations. A p-value less than or equal to 0.05 was deemed significant.

**Results:**

The prevalence of poor sleep quality (PSQI ≥ 5) was 41.7%, while 18.3% of participants exhibited psychosocial distress, predominantly in the internalizing domain (16.7%). A significant association was found between psychosocial distress and poor sleep quality (*p* = 0.039). Adolescents with psychosocial distress had a 2.10 times higher risk of poor sleep (95% CI: 1.24–3.55). Notably, inpatient status was significantly associated with worse sleep compared to outpatients (*p* = 0.030). PSC-17 scores showed a positive correlation with total PSQI scores (*r* = 0.301, *p* = 0.019) and were specifically linked to the daytime dysfunction dimension (*r* = 0.365, *p* = 0.004).

**Conclusion:**

Poor sleep quality is common among adolescents with cSLE and is significantly associated with psychosocial distress, particularly reflected in daytime dysfunction. These findings support integrating routine screening of sleep quality and psychosocial distress into comprehensive cSLE care and highlight the need for larger longitudinal studies to clarify causal pathways and identify modifiable drivers of sleep disturbance.

## Background

Adequate sleep is vital for physical and mental health, and essential for growth and development in adolescents [1]. Sleep is associated with reduced motor activity, less engagement with the external world, a particular posture (e.g., lying down with closed eyes), and easy reversibility [2]. During adolescence, hormonal changes and pubertal maturation may affect sleep patterns, making this phase vulnerable to poor sleep quality [3, 4]. Social pressures and modern lifestyle factors also contribute to sleep deprivation in adolescents [5]. Moreover, adolescents with chronic illnesses, such as cSLE, are specifically at an increased risk of having poor sleep quality due to the disease’s progression, recurrent symptoms, and prolonged management [6].

The characteristics of SLE are the overproduction of autoantibodies and the creation of immune complexes that harm numerous organ systems [7]. Childhood-onset SLE is more progressive than that of adult-onset SLE [7–10]. It accounts for 15–20% of all SLE patients, with an incidence of 0.36 to 2.5 per 100,000 children and a prevalence ranging from 1.89 to 34.1 per 100,000 children [7, 8]. Sleep disorders and poor sleep quality are frequently reported in the adult SLE population [6, 11, 12], but the causes of poor sleep quality have not been identified and are highly complex, modulated by various factors [6, 12]. Behavioural and psychosocial factors, such as physical activity, depression, and stress, influence sleep quality [12, 13], with the relationship between sleep quality and its causative factors considered bidirectional [6, 12].

Sleep is particularly critical in pediatric lupus, as it is closely involved in immune regulation, hormonal balance, and neurodevelopment—processes that are still maturing during childhood and adolescence and may be further disrupted by chronic inflammation [1, 2]. Adolescents with cSLE have been reported to experience a higher prevalence of sleep disturbances compared with healthy peers, which may contribute to increased disease burden and impaired health-related quality of life [11, 13] Despite limited data in pediatric populations, existing evidence suggests that addressing sleep disturbances in cSLE is essential for comprehensive disease management and long-term outcomes [11, 14, 15].

Cytokine dysregulation and SLE treatment, such as the use of long-term steroids and immunomodulators, are thought to contribute to poor sleep quality in SLE patients [11, 12, 16]. Corticosteroids, the main SLE treatment, are suspected to trigger depression and sleep disorders [1, 2]. Depression is a psychosocial distress frequently reported among SLE patients,^6^ with an estimated 50–90% of patients with psychosocial distress experiencing sleep quality problems [12].

To date, most studies on poor sleep quality in SLE have been conducted in adults; therefore, this study was designed to evaluate the relationship between psychosocial status and sleep quality in cSLE adolescents.

## Method

### Patient selection

This cross-sectional observational study of inpatients and outpatients from the Division of Allergy, Immunology, and Rheumatology, Hasan Sadikin Hospital, Bandung, Indonesia, was conducted from July to August 2025. The inclusion criteria were patients aged 10–18 years old diagnosed with cSLE according to the 2012 Systemic Lupus International Collaborating Clinics (SLICC) criteria, the 2019 European Alliance of Associations for Rheumatology (EULAR)/ACR criteria, or the 1997 American College of Rheumatology (ACR) criteria were eligible for inclusion [17]. Patients had a mild to moderate disease activity level assessed by the MEX-SLEDAI score at the time of assessment, and the patients themselves were required to complete the Pediatric Symptom Checklist-17 (PSC-17) and the Pittsburgh Sleep Quality Index (PSQI) questionnaires, with guidance provided by the research team when needed. Patients were excluded if they exhibited signs of severe illness activity (severe MEX-SLEDAI score) or had a reduced level of awareness (GCS below 14). All patients and parents were provided with the study information and the opportunity to ask questions before providing written informed consent for participation. The research was authorized by the Director of Dr. Hasan Sadikin General Hospital, and ethically approved by the Ethics Committee of Dr. Hasan Sadikin General Hospital (no: DP.04.03/D.XIV.6.5/277/2025).

### Psychosocial status measurement

Screening programs utilize initial assessments and predetermined cut-off points to effectively stratify risk and minimize the occurrence of missed cases, thereby facilitating a streamlined transition to diagnostic confirmation. Several validated screening instruments may be employed for this purpose, including the Pediatric Symptom Checklist (PSC), the Strengths and Difficulties Questionnaire (SDQ), and the Patient Health Questionnaire (PHQ) [18]. Psychosocial status was assessed using the PSC-17 questionnaire, which is a screening instrument recommended by Indonesian Pediatric Society as a primary tool for early detection of psychosocial distress [19]. The PSC-17 questionnaire consists of 17 items designed to help identify and evaluate changes in emotional and behavioral issues among children and adolescents. The questionnaire comprises three domains: internalizing, externalizing, and attention, and is scored using a Likert scale, where 0 means never, 1 means sometimes, and 2 means often experiencing the symptom. The internalizing domain included feelings of sadness, hopelessness, low self-esteem, moodiness, and anxiety. The externalizing domain included not liking to share, lacking empathy, fighting, blaming others, disobeying rules, bothering other children, and taking others’ belongings. The attention domain included restlessness, daydreaming, difficulty concentrating, acting without thinking, and being easily distracted. A patient was considered to have psychosocial distress if they had a score > 5 on the internalizing subscale, > 7 on the externalizing subscale, > 7 on the attention subscale, or a total score of > 15. This questionnaire had previously been translated into Indonesian and demonstrated good validity and reliability. It can be self-administered among adolescents, thereby minimizing observer bias [19, 20].

### Sleep quality measurement

The sleep quality over a one-month interval was assessed using the Indonesian version of the PSQI questionnaire, which was validated for adolescents in Indonesia in 2018 [21]. This sleep measurement tool, containing 19 items, is often used in studies on healthy adolescents and adults with SLE to evaluate sleep latency, length, subjective quality, efficiency, disruptions, medication use, and daytime dysfunction. It is scored using a 5-point Likert scale, with a total PSQI score ranging from 0 (excellent) to 21 (poor), to determine the respondent’s sleep experience and quality during the last month. Good sleep is defined as a PSQI score of 5 or less, while poor sleep is defined as a score of 5 or more [11, 21].

### Patient characteristics

All 60 patients underwent clinical and laboratory evaluation. The data collected included age, sex, nutritional status, disease duration, duration of steroid use, and steroid dose. Nutritional status and height status were classified based on the World Health Organization (WHO) Growth Reference [22]. The steroid dosage was based on the prednisone dose and divided into three categories: no use or low dose (0.1 − 0.2 mg/kg), moderate dose (0.2 − 0.5 mg/kg), and high dose (0.5 − 1 mg/kg) [23]. Disease activity was assessed using the MEX-SLEDAI and classified as mild or moderate [24].

### Statistical analysis

The number of adolescents with cSLE (*n* = 60) was used to calculate the power to detect a difference in the prevalence of a possible risk factor between 20% and 55% exposure. With a 2-sided significance level of 5%, this study would be able to detect this difference with around 80% power.

The data were analyzed using SPSS for Windows, version 25.0, and normality was assessed using the Kolmogorov-Smirnov test. The participants’ characteristics were represented by descriptive statistics and bivariate analysis to determine the relation with sleep quality. The participants’ emotional and mental health, as well as the quality of their sleep, were represented by descriptive statistics. Bivariate analysis using Fisher’s exact test or Chi-Square tests was conducted to determine how psychosocial state relates to sleep quality. Additionally, the data were stratified for a sub-analysis to identify potential variations between inpatient and outpatient settings. The relationship between psychosocial state and the characteristics of sleep quality (PSQI) was assessed using Spearman’s rank correlations. A p-value < 0.05 was considered statistically significant.

## Results

### Patient characteristics

A total of 70 cSLE adolescents were initially assessed for eligibility. Of these, 8 patients were excluded because they did not meet the inclusion criteria, and 2 patients declined to participate. Consequently, 60 adolescents were enrolled and included in the final analysis. This study involved 60 cSLE adolescents, aged 10.3 to 17.9 years, with a median age of 15.5 years. The study population was predominantly female (85%; 51 adolescents). In terms of clinical status, the majority of participants were inpatients (73.3%; 44 adolescents), while 26.7% were managed as outpatients. Based on anthropometric data, most of the adolescents had good nutritional status (70%; 42 adolescents). Most patients presented with mild disease activity according to MEX SLEDAI scores (76.7%; 46 adolescents) and had been receiving steroid treatment for one year or less (66.7%; 40 adolescents). The majority of patients were on high-dose steroids (28 adolescents; 46.7%). Although steroid dose did not reach the threshold for statistical significance (*p* = 0.062), a notable trend was observed: patients on high-dose steroids had a higher prevalence of poor sleep (57.1%; 16 adolescents) compared to others. The bivariate analysis revealed that inpatients status was significantly associated with sleep quality (*p* = 0.030), 50% of inpatients exhibited poor sleep quality (PSQI **≥** 5), whereas only 18.8% of outpatients reported poor sleep, indicating that hospitalized patients are significantly more likely to experience sleep disturbances. The patients’ characteristics and its relationship with sleep quality are shown in Table 1.

**Table 1:** Patient characteristics and association with sleep quality

Variable	Total (*n* = 60)	Sleep Quality		*p*-value
		Bad (PSQI ≥ 5)	Good (PSQI < 5)	
Age (years), Median (min–max)	15.5 (10.3–17.9)	15.3 (10.3–17.9)	15.5 (10.7–17.7)	0.942^c^
Age category, n (%)				0.922^a^
10–12 years (Early Teens)	8 (13.4)	3 (37.5)	5 (62.5)	
13–15 years (Mid Teens)	32 (53.3)	13 (40.6)	19 (59.4)	
16–18 years (Late Teens)	20 (33.3)	9 (45.0)	11 (55.0)	
Sex, n (%)				0.722^b^
Male	9 (15.0)	3 (33.3)	6 (66.7)	
Female	51 (85.0)	22 (43.1)	29 (56.9)	
Patient Status, n (%)				0.030^a^*
Inpatient	44 (73.3)	22 (50.0)	22 (50.0)	
Outpatient	16 (26.7)	3 (18.8)	13 (81.3)	
Nutritional status, n (%)				0.783^a^
Good (BMI-for-Age − 2 SD ≤ x ≤ 1 SD)	42 (70.0)	18 (42.9)	24 (57.1)	
Low (BMI-for-Age < − 2SD)	4 (6.7)	1 (25.0)	3 (75.0)	
Over (BMI-for-Age > 2SD)	14 (23.3)	6 (42.9)	8 (57.1)	
Disease duration, n (%)				0.711^a^
≤ 1 year	40 (66.7)	16 (40.0)	24 (60.0)	
> 1 year	20 (33.3)	9 (45.0)	11 (55.0)	
MEX SLEDAI Score, n (%)				0.470^a^
Mild	46 (76.7)	18 (39.1)	28 (60.9)	
Moderate	14 (23.3)	7 (50.0)	7 (50.0)	
Steroid dose, n (%)				0.062^a^
Low	22 (36.6)	7 (31.8)	15 (68.2)	
Moderate	10 (16.7)	2 (20.0)	8 (80.0)	
High	28 (46.7)	16 (57.1)	12 (42.9)	
Steroid treatment duration, n (%)				0.853^a^
≤ 1 year	40 (66.7)	17 (42.5)	23 (57.5)	
> 1 year	20 (33.3)	8 (40.0)	12 (60.0)	

Note: ^a^Chi-squared test, ^b^Fisher’s exact, ^c^t-test, *p < 0.05

### Psychosocial status

The PSC-17 scores ranged from 0 to 16, with a median of 7. The prevalence of cSLE adolescents who had psychosocial distress was 18.3% (11 individuals) (Table 2). Among cSLE adolescents who showed a tendency toward psychosocial distress, the internalizing domain was the most commonly affected.

**Table 2:** Psychosocial status

Variable	Total (*n* = 60)
PSC 17 Score, median (min–max)	7 (0–16)
Psychosocial Status, n (%)	
Psychosocial Distress	11 (18.3)
Normal	49 (81.7)
Internalizing Domain, n (%)	
Internalizing problems	10 (16.7)
Normal	50 (83.3)
Attention Domain, n (%)	
Attention problems	1 (1.7)
Normal	59 (98.3)
Externalizing Domain, n (%)	
Externalizing problems	0 (0.0)
Normal	60 (100.0)

### Sleep quality

The median PSQI score in the overall cohort was 4 (range, 0–12) A total of 25 adolescents (41.7%) had a PSQI score of **≥** 5, indicating poor sleep quality, whereas 35 adolescents (58.3%) had a PSQI score of < 5. The median and range for each component PSQI are presented in Table 3.

**Table 3:** Sleep quality

Variable	Total (*n* = 60)	Bad (PSQI ≥ 5) (*n* = 25)	Good (PSQI < 5) (*n* = 35)
PSQI Score, median (min–max)	4 (0–12)	5 (5–12)	2 (0–4)
Component, median (min–max)			
Subjective sleep quality	0.62 (0–2)	1.00 (0–2)	0.34 (0–2)
Sleep latency	0.78 (0–3)	1.28 (0–3)	0.43 (0–2)
Sleep duration	0.52 (0–3)	0.96 (0–3)	0.20 (0–2)
Sleep efficiency	0.27 (0–3)	0.60 (0–3)	0.03 (0–1)
Sleep disturbances	1.03 (0–3)	1.60 (0–3)	0.63 (0–3)
Daytime dysfunction	0.57 (0–2)	0.96 (0–2)	0.29 (0–1)

Note: All participant did not have the use of Sleeping medication

### Psychosocial status and sleep quality

The relationship between psychosocial status and sleep quality in cSLE adolescents was statistically significant (*p* = 0.039). Adolescents who were having psychosocial distress had a higher proportion of poor sleep quality (72.7%) compared to those with a normal psychosocial status (34.7%). Prevalence ratio analysis indicated that adolescents with psychosocial distress had a 2.10 times higher risk of poor sleep quality compared to the normal group (95% CI: 1.24–3.55) (Table 4). When stratified by patient status, distinct patterns emerged. Within the inpatient cohort, although the prevalence of poor sleep was higher among those with psychosocial distress (70%) compared to those with normal status (44.1%), this association did not reach statistical significance (*p* = 0.281; PR: 1.58; 95% CI: 0.91–2.76). In contrast, a significant association was observed among outpatients (*p* = 0.032). Remarkably, all outpatients identified with psychosocial distress (100%) reported poor sleep quality. This resulted in a substantial prevalence ratio, with outpatients with psychosocial distress being 7.50 times more likely to have poor sleep (95% CI: 2.05–27.25).

**Table 4:** Association between psychosocial status and sleep quality

Variable	Sleep Quality		*P*-value	PR (95% CI)
	Bad PSQI ≥ 5	Good PSQI < 5		
**Psychosocial Status**				
Psychosocial Distress	8 (72.7)	3 (27.3)	0.039^b^*	2.10 (1.24–3.55)
Normal	17 (34.7)	32 (65.3)		
** Inpatient**				
Psychosocial Distress	7 (70.0)	3 (30.0)	0.281^b^	1.58 (0.91–2.76)
Normal	15 (44.1)	19 (55.9)		
** Outpatient**				
Psychosocial Distress	1 (100)	0 (0)	0.032^a^*	7.50 (2.05–27.25)
Normal	2 (13.3)	13 (86.7)		

Note: ^a^Pearson chi-squared test, ^b^Fisher’s exact test, *p < 0.05

There was a positive correlation between psychosocial status scores and total PSQI scores (*r* = 0.301; *p* = 0.019), indicating that higher PSC-17 scores, reflecting a tendency toward psychosocial distress, were associated with poorer sleep quality (Table 5). In the analysis of PSQI dimensions, psychosocial status scores were only significantly associated with the daytime dysfunction dimension (*r* = 0.365; *p* = 0.004).

**Table 5:** Correlation between psychosocial status and sleep quality

Variable	Psychosocial Status (PSC-17)	
	Spearman coefficient	*p* Value
**Sleep quality (PSQI)**	0.301	0.019*
**Component**		
Subjective sleep quality	0.182	0.164
Sleep latency	0.171	0.192
Sleep duration	0.189	0.148
Sleep efficiency	0.235	0.070
Sleep disturbances	0.199	0.127
Daytime dysfunction	0.365	0.004*

Note: Spearman’s rank correlations, *p < 0.05; No participants used sleep medication

## Discussion

This study demonstrates that poor sleep quality is common among cSLE adolescents, affecting nearly half of the adolescents, and is significantly associated with psychosocial distress. Adolescents with psychosocial distress had more than twice the risk of poor sleep quality compared with those without such problems, and higher PSC-17 scores were positively correlated with worse overall sleep quality. The Indonesian version of PSC-17 is a valid and highly reliable instrument for detecting psychosocial distress in adolescents19, and indicated that 18.3% of cSLE adolescents in this study had psychosocial distress. It has been reported that children with rheumatologic diseases in children often experience mental health issues such as anxiety and depression compared to their healthy peers [14]. A high prevalence of depression has also been reported in cSLE patients [25]. However, studies conducted in Southeast Asia reported that a third of cSLE patients had a risk of depression [11, 26, 27], more than observed in the present study. Internalizing problems were found to be more predominant and are one of the symptoms of psychosocial distress in children and adolescents identified by the PSC-17 [19]. Internalizing problems are described as a tendency towards anxiety, depression, nervousness, and social withdrawal [19].

The lower prevalence of depression observed in this study (13%), compared with other Southeast Asian childhood-onset SLE cohorts—such as the approximately 32% reported in a Thai pediatric lupus clinic—may be influenced by cultural stigma and reluctance to disclose psychological symptoms. Such underreporting may be further compounded by limited mental health literacy and restricted access to youth-friendly mental health services, which can reduce help-seeking behavior and hinder case detection among adolescents with cSLE [27, 28]. Additionally, variations in healthcare context and methodological approaches across studies, including differences in screening instruments, cutoff values, and the routine implementation of standardized screening and referral pathways, may substantially affect reported prevalence estimates [14]. Inadequate or inconsistent mental health screening has been shown to miss clinically relevant depressive symptoms in pediatric SLE care, potentially leading to an underestimation of true disease burden [14, 28].

Consistent with these findings Neufeld also reported that a cSLE population had a high frequency of symptoms of depression (35%) and anxiety (35%) [28]. The stress of living with a chronic condition is one of the contributing factors to anxiety and depression symptoms in cSLE, as they found no relationship between factors related to SLE, such as disease duration, disease activity, or steroid use, and anxiety and depressive symptoms [28]. Chronic psychosocial stress can cause sub-threshold symptoms to develop into diagnostic symptoms in vulnerable individuals, increasing the risk of future mental health disorders, especially in developing adolescents [28]. In contrast, Puspitanza reported that disease activity and low physical activity were related to depression [26]. Low physical activity in SLE patients is linked to an increase in pro-inflammatory cytokines and an imbalance of hormones and neurotransmitters in the brain, such as serotonin and dopamine, which can cause mood disorders, especially depression, in cSLE patients [26].

Given the relatively limited literature examining sleep quality in cSLE adolescents, our findings highlight sleep disturbances as a prominent clinical concern, with a substantial proportion of patients demonstrating poor sleep quality. In the present study, nearly half of the adolescents reported poor sleep quality based on their PSQI scores [21]. The high prevalence of poor sleep quality among SLE patients (56–60%) is often underrecognized, undertreated, and understudied [12]. In the SLE adult population, complaints of difficulty initiating or maintaining sleep, as well as poor sleep quality, are frequently reported [11–13]. According to Wi et al., cSLE adolescents have poorer sleep quality compared to healthy adolescents, measured by both objective actigraphy and subjective sleep measurements, supporting the current findings regarding poor sleep quality in cSLE adolescents [15].

Many factors influence sleep quality in SLE patients, including disease duration, medication side effects, and comorbidities conditions like arthralgia, pain, fatigue, and depression [29]. Yottasan et al. reported a relationship between poor sleep quality and a high body mass index, which differs from the current study, in which the anthropometric status of adolescents was not related to sleep quality; however, their study sample contained more overweight adolescents (38.6% vs. 23.3%).^11^ Short sleep duration affects body weight, especially in adolescents who are in a crucial period of life, obtaining independence, enhancing their social life, and having a high desire to adapt to a modern lifestyle [30]. Additionally, they may experience pressure due to a high study load and homework, all cumulatively resulting in reduced sleep time, fatigue, and physical activity, and ultimately increasing the consumption of energy-dense food during waking hours [30]. These social and behavioral factors complement previously hypothesized mechanisms, such as hormonal dysregulation, changes in body metabolism, daytime sleepiness, and fatigue, which lead to lack of sleep, resulting in increased energy consumption [30].

In this study steroid dosage was not significantly associated with sleep quality in this study (*p* = 0.062), the proximity of this value to the conventional significance threshold suggests a potential trend that may have reached statistical significance with a larger sample size. Yottasan et al. also reported that steroid exposure did not affect sleep quality, similar to the current study [11]. This is in contrast to observations in the adult SLE population, in which SLE medication is related to sleep quality [16]. Corticosteroids are known to affect sleep regulation through their influence on circadian rhythm, mood, and hypothalamic–pituitary–adrenal axis activity, and have been frequently implicated in sleep disturbances among patients with systemic lupus erythematosus [12, 16]. Özer et al. also reported that poor sleep quality worsened in patients with a longer disease duration [16]. This differs from the current results, where disease duration did not have a significant relationship with sleep quality. In SLE, an increase in the duration of the disease increases the possibility of the number of organs involved, aggravating symptoms, and thus affecting the patient’s sleep quality [16]. The average good sleep quality in the first year of the disease was a finding in Özer’s study [16]. Although pediatric-specific evidence remains limited, sleep deficiency in children and adolescents with cSLE has been associated with increased pain, fatigue, and depressive symptoms, suggesting that medication-related sleep effects may contribute indirectly to disease burden [15].

In the present study, there was a weak correlation between psychosocial distress and sleep quality, in line with Yin’s comprehensive research, which discovered a modest association between the quality of sleep and depression in individuals with SLE [6]. Mental health issues, including depression, have been linked to issues with subjective sleep quality, latency, disruption, and daytime functioning [6]. Other aspects of sleep, including the amount of time spent sleeping, sleep quality, and whether or not medicine was used to aid with sleep, did not show any association [6]. Slightly different from the present study results, the findings show that only the daytime dysfunction component was significantly correlated with psychosocial status. The underlying mechanism of poor sleep quality is not clear. However, sleep deprivation may disrupt the circadian rhythm by impacting the brain regions responsible for emotions, the amygdala and the anterior cortex, which are also involved in sleep regulation. This dysregulation occurs during depressive episodes [6].

Tangkittiwet et al. used the Patient Health Questionnaire for Adolescents (PHQ-A) and the Generalized Anxiety Disorders Scale (GAD-7) to evaluate symptoms of anxiety and sadness, suggesting that there is a link between cSLE patients’ anxiety and depressive symptoms and the quality of their sleep [27]. The correlation in their study was moderate, while the current study’s correlation was weak. Wi et al. further reinforced the relationship between depression and poor sleep in the cSLE population [15]. Functional connectivity in many brain areas, such as the lateral orbitofrontal cortex, dorsolateral prefrontal cortex, cingulate cortex, and precuneus, is positively correlated with poor sleep quality and depressive symptoms [31]. Functional connectivity between these areas contributes significantly to the relationship between depressive disorder and poor sleep quality [31], as sleep-wake homeostasis is mediated by the nervous system in the brainstem and hypothalamus with the cerebral cortex.

This study has several limitations. In the study, only bivariate relationships were examined with a minimal sample size, which limited the multivariate analysis between the existing variables. The other limitation is the inclusion of both inpatients and outpatients introduces a potential selection bias. Hospitalized patients often experience acute psychological stress and environmental disruptions (such as overnight clinical monitoring and hospital noise) that can independently impair sleep quality. While the PSQI assesses the previous month’s sleep, the immediate experience of hospitalization may have influenced patient recall or current perceptions. Furthermore, this study did not account for several clinical confounders, including pain, fatigue, and the presence of comorbid conditions, which are known to independently affect sleep quality. Future research should employ a more comprehensive set of clinical metrics to better isolate the primary drivers of sleep disturbance in this population. Finally, there was no further evaluation to determine the definitive diagnosis of patients with psychosocial distress.

## Conclusion

This study demonstrates that adolescents with childhood-onset systemic lupus erythematosus (cSLE) exhibit a notable prevalence of psychosocial distress, particularly within the internalizing domain, alongside a substantial burden of poor sleep quality. Although only a weak correlation was observed between psychosocial distress and sleep quality, the findings highlight the vulnerability of cSLE adolescents to coexisting psychological and sleep disturbances, emphasizing sleep as a clinically relevant yet relatively underexplored domain in pediatric lupus care. As PSC-17 and PSQI function primarily as screening tools, these findings should be interpreted as indicators of potential risk rather than diagnostic outcomes, underscoring the need for comprehensive mental health evaluation. Future studies with larger cohorts, longitudinal designs, and broader clinical variables are warranted to clarify causal pathways and to better define the role of psychosocial factors in sleep disturbances among adolescents with cSLE.

## Data Availability

No datasets were generated or analysed during the current study.

## Abbreviations

cSLE: Childhood-onset Systemic Lupus Erythematosus
PSQI: Pittsburgh Sleep Quality Index
PSC-17: Pediatric Symptom Checklist-17
SLICC: Systemic Lupus International Collaborating Clinics
EULAR: European Alliance of Associations for Rheumatology
ACR: American College of Rheumatology
WHO: World Health Organization
PedsQL: Pediatric Quality of Life Inventory
PHQ-A: Patient Health Questionnaire for Adolescents
GAD-7: Generalized Anxiety Disorder Scale
